# Ultra-processed Foods, Weight Gain, and Co-morbidity Risk

**DOI:** 10.1007/s13679-021-00460-y

**Published:** 2021-10-22

**Authors:** Anthony Crimarco, Matthew J. Landry, Christopher D. Gardner

**Affiliations:** Stanford Prevention Research Center, School of Medicine, Stanford University, Palo Alto, CA USA

**Keywords:** Ultra-processed foods, Weight gain, NOVA, Weight management, Chronic disease

## Abstract

***Purpose of Review*:**

The purpose of this review is to provide an update on the available data regarding the associations of Ultra-processed food (UPF) consumption with food intake and possible underlying mechanisms relating UPF consumption to weight gain and co-morbidities.

***Recent Findings*:**

In primarily observational studies, UPF consumption is consistently associated with an increased risk for weight gain among adults and children and increased risk for adiposity-related co-morbidities in adults. In a single mechanistic study, consumption of UPFs led to increased energy intake and weight gain relative to whole foods.

***Summary*:**

UPFs tend to be more energy-dense than nutrient-dense, and UPF consumption is associated with increased adiposity and co-morbidity risk. These data suggest that recommendations to limit UPF consumption may be beneficial to health — though further mechanistic studies are needed.

## Introduction

Food processing includes any variety of operations to modify and alter raw foods from their natural state to make them more suitable for consumption, cooking, or storage [1]. This includes heating, freezing, washing, fermentation, grinding, packaging, and other operations. Since prehistoric times, our ancestors mastered the use of fire for the purpose of heating and cooking foodstuffs to preserve their organoleptic and nutritional properties [2]. During more recent historical events like the Industrial Revolution or the second World War, the focus on food processing began to shift from home cooking to more industrialized processes to emphasize the preservation, safety, and nutritional quality of foods [2].

Food processing has been integral to providing safe, edible, and nutritious foods to the population for centuries. It is useful for increasing the shelf life of foods, optimizing nutrient availability and food quality, as well as to reduce losses and waste [3, 4]. Since the nineteenth century, a number of technologies in food processing were introduced, including canning and pasteurization. This was followed by many types of physical, thermal, and chemical processes, such as centrifugation and sterilization of dairy products, or bleaching vegetable oils [5]. The topic of food processing is complex, and the different types of processes bring both benefits and risks. For example, heat processing increases the shelf life and decreases the pathogenic potential of raw milk, but promotes the loss of nutritional value or the production of mutagenic or carcinogenic molecules in others [2, 6]. Thus, different types of food processing bring both benefits and risks.

Advancements in food processing and changes to our agro-industrial systems have led to the development of numerous food products that contribute to the so-called “Westernized diet.” These Westernized food products are usually highly processed and energy-dense, and they contain high amounts of added sugar, saturated fat, and salt, but low amounts of fiber [7]. The concerns about the health effects of industrial processing on diet quality and chronic disease risk has resulted in food classification systems to distinguish between different categories of processed foods [8]. The most popular of those food classification systems is the NOVA (not an acronym) system, which introduced the term “ultra-processed foods” to describe the highest level of food processing [8, 9]. Ultra-processed foods (UPFs) tend to be highly palatable, convenient, shelf stable, and affordable, and are often marketed and advertised in appealing ways [7, 10–13]. Since the term UPFs was coined, there were 72 articles published on the subject between 2009 and 2016 and another 565 articles from 2017 and 2021 (based on a PubMed search of title words). The increased focus on the health effects of UPFs has resulted in a number of studies assessing the association between UPFs with weight gain and/or co-morbidity risk.

There have also been recent studies that documented an increased consumption of UPFs during the shelter-in-place lockdowns that were implemented to prevent the spread of the novel coronavirus (COVID-19) [14–16]. This was largely attributable to an increase in fast foods and the consumption of low-quality meals or snacks, such as sweets, chocolates, sugar-added beverages, and processed meat. A recent review indicated that only one study showed any improvement in food quality intake (i.e., increased fruit and vegetable consumption) among participants during the shelter-in-place period [16].

Based on the growing interest and potential concerns about the adverse health effects associated with consuming UPFs, the purpose of this review is to examine recent literature (i.e., within the last 5 years) on UPF consumption and its association with weight gain and/or co-morbidity risk. We also discuss the potential mechanisms of how UPFs increase the risk of gaining weight and developing chronic diseases, as well as the limitations of the NOVA classification system.

## Defining Ultra-Processed Foods

In food science and technology, the level of food processing is based on the intensity and amount of operations utilized to enhance shelf life, food safety, food quality, and availability of edible parts of raw materials [17, 18]. There are numerous definitions of food processing from organizations like the International Food Information Council (IFIC) [19] or the International Agency for Research on Cancer—European Prospective Investigation into Cancer (IARC-EPIC) [20]. In general, these classification systems were designed by researchers to study the relationships between industrial products and nutritional intake and/or chronic disease risk [3]. The United States Department of Agriculture (USDA) defines a processed food as “any raw agricultural commodity that has been subject to washing, cleaning, milling, cutting, chopping, heating, pasteurizing, blanching, cooking, canning, freezing, drying, dehydrating, mixing, packaging, or other procedures that alter the food from its natural state” [21].

**Fig. 1:**
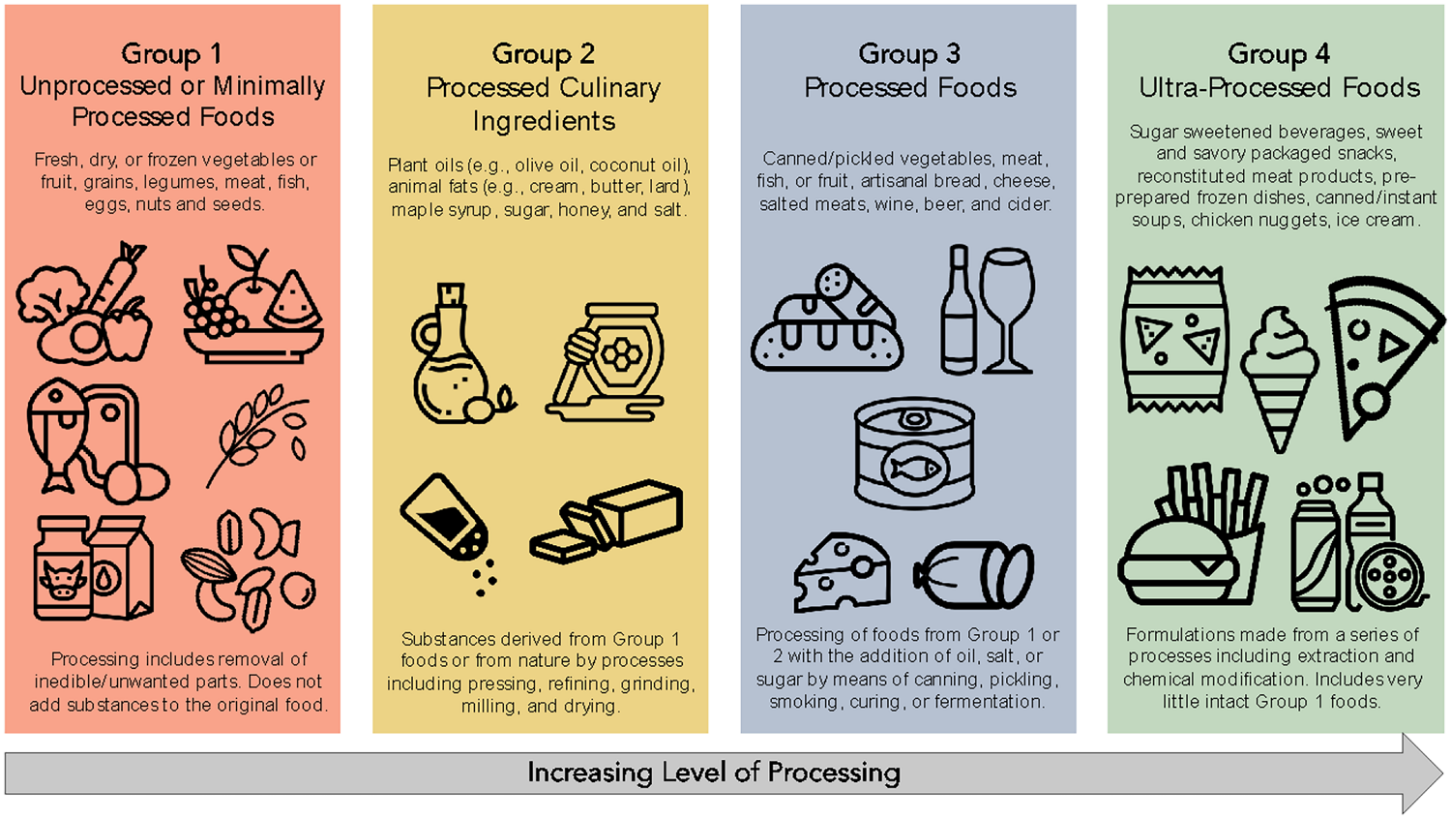
Spectrum of processing of foods based on the NOVA classification. The figure provides examples of foods and types of processing methods within each NOVA classification group. Definitions are adapted from Monteiro et al. (2018) [8]

The NOVA system is one of the most popular food classification systems for categorizing foods and beverages in the public health literature. One of the first systematic reviews on food processing that was published in this journal concluded that NOVA was the most specific, coherent, clear, comprehensive and workable definition [22]. The NOVA criteria involve classifying food products into four groups based on the amount of processed ingredients: (1) unprocessed or minimally processed foods, (2) processed culinary ingredients, (3) processed foods, and (4) ultra-processed foods (UPFs) [9]. See Fig. 1 for the complete definitions of all food categories. The UPF category is described as “formulations mostly of cheap industrial sources of dietary energy and nutrients plus additives, using a series of processes” [9]. Some examples include reconstituted meats, frozen pizzas, and confectionary foods, to name a few. The concept of UPFs was originally coined and developed by a team from the University of São Paulo in a 2009 commentary [8]. The main argument of the commentary was that the extent to which foods are processed, rather than specific nutrients or food items, is the most important factor for determining the relationship between nutrition and chronic diseases. This work has now been formally adopted as a part of the national dietary guidelines in Brazil [23] and has been acknowledged in several leading reports, such as the Food and Agriculture Organization (FAO) of the United Nations [24] or the Pan American Health Organization (PAHO) of the World Health Organization (WHO) [25].

Because the food manufacturing industry is not required to state the processes used in its products on food labels and the information required on food labels is not standardized across countries, it can be difficult for consumers to identify UPFs easily [7, 26]. For example, products like plain steel-cut oats, plain corn flakes, and shredded wheat are classified as *minimally processed* foods, but the same foods are considered *processed* when they also contain sugar, and *ultra-processed* if they also contain flavors or colors [7]. A general rule of thumb is to identify food substances or additives whose primary function is to make the final product more palatable or more appealing (i.e., “cosmetic additives”). This includes items like hydrolyzed proteins, high-fructose corn syrup, and interesterified oils, to name a few.

Because classification systems like NOVA largely rely on categorizing a processed food category based on the content of added sugars, saturated fat, and sodium, it is possible to misclassify some nutrient-rich foods as ultra-processed [27]. For example, in Drewnowski et al.’s analysis of various foods using both the Nutrient Rich Food and NOVA criteria, fortified ready-to-eat cereals, as well as beans and nuts (in the form listed in the FFQ), were classified as ultra-processed from the NOVA definitions [27]. Additionally, when conducting diet assessments for research purposes (e.g., food frequency questionnaires, 24-h recalls), it is not typical to specify the level of processing involved in reported foods, and thus misclassification may also result here [28]. For example, using the NOVA classification system, commercially baked bread has been classified as *ultra-processed*, whereas the same bread was considered *processed* when homemade [29]. Foods that are processed by innovative, non-traditional techniques, such as electric or magnetic fields, may be deemed as *minimally processed*, despite the use of non-traditional, complex processes [30–32]. Some of the guidelines on UPFs may imply that food processing as a concept has a negative connotation. Sadler et al. [3] note that this could potentially “encourage consumers to seek out unprocessed foods (e.g. raw milk) or process foods at home, without sufficient food safety controls, and such consumer rejection could also hamper sustainable innovations” [3].

More terms and definitions have recently been added to address some of the classification problems within the original NOVA criteria. The Siga classification of processed foods extends the NOVA classification system by combining the original 4 categories of food processing with 5 more specific subgroups [33]. This classification system accounts for added sugar, fat, and salt contents; “at risk” additives; “matrix” effects; ultra-processed ingredients; and the number of markers of ultra-processing (MUPs). Most of the literature to date still utilizes the original NOVA criteria; there have not yet been many studies published utilizing the Siga criteria [33].

## Ultra-Processed Food Consumption Levels

UPFs are expanding in food systems across the globe. A number of articles have been published on the contribution of UPF consumption to daily total energy intake in different countries. Table 1 shows selected recent articles on the subject. In general, the majority of calories consumed in high-income countries are from ultra-processed foods and beverages. For example, in Canada, the UK, and the USA, UPF products were estimated to contribute 45.0%, 50.4%, and 57.9% of total energy intake, respectively [34–36]. For other countries like Brazil, UPFs contributed 22.7% of total energy intake. For other countries like Brazil, UPFs contributed 22.7% of total energy intake. But it should be noted that older studies (not shown in Table 1) have indicated that UPFs contributed anywhere from 21.5 to 51.2% of total energy intake, depending on the sample [37, 38]. Most samples included children and adults, while two focused only on children [39, 40]. Monteiro et al. (2018)  assessed household availability of NOVA food groups in 19 European countries and analyzed the association between availability of UPF and prevalence of obesity [35]. A strength of the study was the use of standardized data and the use of population-based, actual (non-modeled) estimates of the prevalence of obesity. After adjusting for multiple confounding factors, each percentage point increase in the household availability of UPF resulted in an increase of 0.25 percentage points in obesity prevalence.

**Table 1:** Select recent articles on ultra-processed food consumption levels from various countries

Articles	Country	Sample	Key findings
Machado et al. [102]	Australia	12,153 individuals from the National Nutrition and Physical Activity Survey (2011–2012) ages 2 years and above	Consumption of ultra-processed foods consisted of 42.0% of total energy intake
Harris et al. [103]	Barbados	Nationally representative population-based sample of 364 adult Barbadians	Consumption of ultra-processed foods consisted of 40.5% of total energy intake
Simões et al. [45]	Brazil	14,378 adults ages between 35 and 74 years sampled at multicenter cohort from 6 public universities	Consumption of ultra-processed foods consisted of 22.7% of total energy intake
Nardocci et al. [34]	Canada	9363 adults ages 18 years or more from the 2004 Canadian Community Health Survey	Consumption of ultra-processed foods consisted of 45.0% of total energy intake
Cediel et al. [49]	Chile	4920 individuals ages 2 years and above	Consumption of ultra-processed foods consisted of 28.6% of total energy intake
Cornwell et al. [39]	Colombia	223 children ages 5–12 years	Consumption of ultra-processed foods consisted of 34.4% of total energy intake
Monteiro et al. [35]	Multiple European countries	Households from the Living Costs and Food Survey (LCFS) or the Data Food Networking (DAFNE)	Consumption of ultra-processed foods consisted of 26.4% of total energy intake. The range of calories consumed from ultra-processed foods were 10.2% of total energy intake in Portugal to 50.4% of total energy intake from the UK
Setyowati et al. [104]	Indonesia	Children and adults (*n* = 145,360) grouped into the following age groups: 0–4, 5–12, 13–18, 19–55, and > 55 years	Consumption of ultra-processed foods consisted of 15.7% of total energy intake
Marrón-Ponce et al. [44]	Mexico	10,087 individuals from the 2012 Mexican National Health and Nutrition Survey	Consumption of ultra-processed foods consisted of 29.8% of total energy intake
Fangupo et al. [40]	New Zealand	669 children ages 1–5 years born in Dunedin, New Zealand	Consumption of ultra-processed foods consisted of 45.0%, 42.0%, and 51.0% of energy intake to the diets of children at 12, 24, and 60 months of age, respectively
Steele et al. [36]	USA	9317 individuals ages 1 year and above	Consumption of ultra-processed foods consisted of 57.9% of total energy intake

**Fig. 2:**
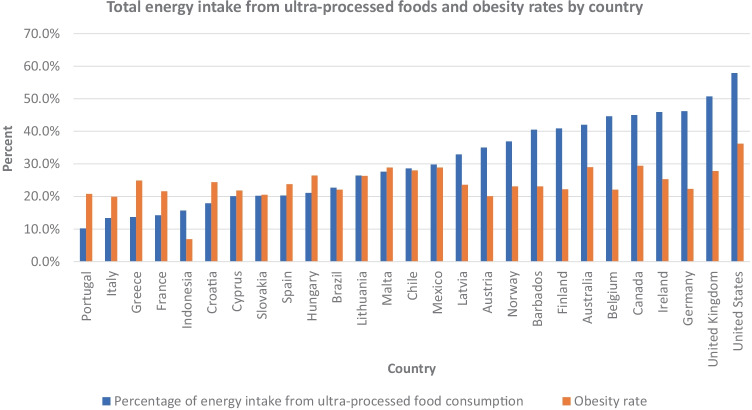
Comparison of select countries totally energy intake from UPFs and obesity rates. Colombia and New Zealand were not included in the graphs, since those studies were based on children only. The obesity rates are based on 2016 data by the World Health Organization (WHO) [41]. Therefore, the rates are not necessarily equivalent to the dietary data from the selected articles

UPF consumption levels also appear to coincide with obesity rates in some of the countries (Fig. 2) [41]. For example, the USA and UK had the highest rates of UPF consumption and obesity. However, for other countries there are inconsistencies. Portugal had a relatively low UPF consumption rate (10.2%), but still has an obesity rate of 20.8%, which is comparable to other European countries [41]. Although UPFs are a significant source of energy intake, they are just one group of foods among all the possible sources of energy intake in the diet, and other factors also contribute to obesity rates.

## Sociodemographic Characteristics

Prior studies have consistently shown that UPF consumption differs among strata of sociodemographic characteristics [34, 42–49]. Differences in consumption vary by gender, age, ethnicity, education, children within the household, nativity, and time for meal preparation depending on the sample (Table 2). The literature has also suggested that an individual’s or a household’s socioeconomic status may be an important factor associated with the consumption of greater ultra-processed foods; however, these associations vary by a country’s income level. Globally, differences between countries can be attributable to differences in food price, affordability, and accessibility [50]. Within the USA, higher consumption of UPF is associated with lower income and education, and studies have documented that UPF account for a larger proportion of grocery spending within households participating in Supplemental Nutrition Assistance Program compared to households not participating [51–53]. Similar results have been found within other high-income countries [42, 47, 54]. The opposite is observed within middle- and low-income countries as those of higher socioeconomic status, those living within urban centers, and those with greater educational attainment are associated with greater UPF intakes [43, 44, 49]. In middle- and low-income countries, there is likely to be more subsistence farming, where families eat more of the food they grow or raise; these foods would be more likely to be whole foods. Those with higher incomes within these countries deviate from traditional dietary patterns as they can afford the purchase of more Westernized foods that are more likely to be processed or ultra-processed.

**Table 2:** Select recent studies examining sociodemographic factors associated with greater consumption of ultra-processed foods

Article	Country	Sociodemographic Factors Associated with Greater Consumption of UPF
Calixto Andrade et al. [105]	France	• Younger age • Urban
Djupegot et al. [42]	Norway	• Men • Native Norwegian • Lower educational attainment • ≥ 3 children within the home • Younger age
Nardocci et al. [42]	Canada	• Men • Younger age • Lower educational attainment • Smokers • Physically inactive • Canadian-born individuals
Khandpur et al. [43]	Columbia	• Younger age • Higher socioeconomic status • Area of residence/geographic region • Urban
Marrón-Ponce et al. [44]	Mexico	• Younger age • Urban • Higher socioeconomic status • Lower educational attainment • Geographical region
Machado et al. [48]	Australia	• Younger • Australian or English country • Physically inactive • Smoker • Lower educational attainment • Urban
Cediel et al. [49]	Chile	• Younger • Urban • Geographic region • Higher income
Baraldi et al. [53]	USA	• Non-Hispanic Whites and Non-Hispanic Blacks (compared to other race/ethnicity groups) • Higher educational attainment • Younger age • Lower income level

## Associations of Ultra-Processed Foods with Weight Gain and Disease Risk in Adults

Although the majority of studies on the health effects of UPFs are observational in nature, there is growing evidence that they contribute to weight gain and increase the risk for some chronic diseases [55]. A meta-analysis of 43 studies (21 cross-sectional, 19 prospective cohort, 2 case–control, and 1 conducted both as a cross-sectional and prospective analysis) indicated that the consumption of UPFs was associated with an increased risk of overweight (odds ratio: 1.36; 95% confidence interval [CI], 1.23–1.51; *P* < 0.001), obesity (odds ratio: 1.51; 95% CI, 1.34–1.70; *P* < 0.001), abdominal obesity (odds ratio: 1.49; 95% CI, 1.34–1.66; *P* < 0.0001), all‐cause mortality (hazard ratio: 1.28; 95% CI, 1.11–1.48; *P* = 0.001), and metabolic syndrome (odds ratio: 1.81; 95% CI, 1.12–2.93; *P* = 0.015) in adults [56]. A recent systematic review of 23 studies (10 cross-sectional and 13 prospective cohort) also found an association between UPF consumption and an increased relative risk for overweight/obesity (+ 39%), high waist circumference (+ 39%), low HDL-cholesterol levels (+ 102%), and metabolic syndrome (+ 79%) [57]. An analysis on a subsample of adult men and women from the PREDIMED-Plus cohort with obesity and metabolic syndrome indicated that higher UPF consumption was associated with greater accumulation of visceral fat, android-to-gynoid fat ratio, and total body fat [58].

A number of other recent reviews that assessed UPF consumption on weight gain, or increased health risk, have reported similar findings (Table 3). The majority of these studies are cross-sectional; therefore, while increased UPF consumption tends to be evident in people with greater adiposity and co-morbidities, the nature of the study design does not indicate any direction of causality. It is worth noting that obesity is a multifactorial disease with many related lifestyle contributors. Given the majority of research on UPFs is observational in nature, residual confounding is possible.

**Table 3:** Narrative reviews, systematic reviews, and meta-analyses summarizing recent evidence evaluating the association between ultra-processed food (UPF) intake and health outcomes

Reference	Type of review	Articles reviewed and study design	Outcome	Key findings
Askari et al. [70]	Systematic review and meta-analysis	13 cross-sectional, 1 prospective cohort	Excess body weight and obesity	UPF consumption was associated with increased risk of overweight and obesity
Chen et al. [71]	Systematic review	8 cross-sectional, 12 prospective cohort	Any health outcome	UPF consumption was associated with increased risk of all-cause mortality, overall cardiovascular diseases, coronary heart diseases, cerebrovascular diseases, hypertension, metabolic syndrome, overweight and obesity, depression, irritable bowel syndrome, overall cancer, postmenopausal breast cancer, gestational obesity, adolescent asthma and wheezing, and frailty. No association with cardiovascular disease mortality, prostate and colorectal cancers, gestational diabetes mellitus, or gestational overweight
Costa et al. [106]	Systematic Review	5 interventions, 6 cross-sectional, and 15 prospective cohorts	Body fat (during childhood and adolescence)	Consumption of UPF was positively associated with body fat during childhood and adolescence
de Miranda et al. [107]	Narrative review	1 randomized controlled trial, 15 prospective cohort, 12 cross-sectional, 1 prospective and cross-sectional, and 1 ecological	Metabolic health	Consumption of UPF was positively associated with metabolic syndrome, body weight change and obesity indicators, blood pressure and hypertension, glucose profile, insulin resistance and type 2 diabetes, other metabolic risks and cardiovascular diseases and mortality
Elizabeth et al. [55]	Narrative review	1 randomized controlled trial, 1 case–control, 19 prospective cohort, 19 cross-sectional, 3 ecological	All health outcomes	Consumption of UPF was positively associated with overweight, obesity and cardio-metabolic risks; cancer, type 2 diabetes and cardiovascular diseases; irritable bowel syndrome, depression and frailty conditions; and all-cause mortality in adults. Among children and adolescents, UPF consumption was associated with included cardio-metabolic risks and asthma
Lane et al. [56]	Systematic Review and Meta-Analysis	21 cross-sectional, 19 prospective, 2 case–control, 1 prospective and cross-sectional	Noncommunicable disease risk, morbidity, and mortality	Consumption of UPF was associated with increased risk of overweight, obesity, abdominal obesity, all-cause mortality, metabolic syndrome, cardiometabolic diseases, frailty, irritable bowel syndrome, functional dyspepsia, breast and overall cancer, depression, and wheezing in adults. Associated with metabolic syndrome in adolescents and dyslipidemia in children. No association with asthma in adolescents
Moradi et al. [108]	Systematic review and meta-analysis	9 cross-sectional and 3 prospective cohort	Overweight, obesity, and abdominal obesity	UPF consumption was associated with increase in the risk of overweight, obesity, and abdominal obesity
Pagliai et al. [57]	Systematic review and meta-analysis	10 cross-sectional and 13 prospective cohort	Any health indicator	Highest UPF consumption associated with increase in the risk of overweight/obesity, high waist circumference, low HDL, metabolic syndrome, all-cause mortality, increased risk of CVD, cerebrovascular disease, and depression. No association with hypertension, hyperglycemia, or hypertriglyceridemia
Santos et al. [109]	Systematic Review	9 cross-sectional and 2 prospective cohort	Cardiometabolic risk factors	UPF were positively associated with overweight and obesity, high blood pressure, and metabolic syndrome
Silva et al. [110]	Systematic Review	13 cross-sectional, 6 prospective cohort, and 2 ecological	Noncommunicable chronic diseases	UPF was positively associated with excess body weight, hypertension, dyslipidemia, and metabolic syndrome

Some prospective cohort studies have also reported that UPFs are positively associated with multiple indicators of adiposity (i.e., BMI, waist circumference, and body fat percent) [59, 60]. A retrospective cohort study indicated that diets rich in UPFs were associated with a 79% increased risk for obesity (HR 1.79; 95% CI 1.06─3.03) and a 30% increased risk for abdominal obesity (HR 1.30; 95% CI 1.14─1.48 [59]. Additionally, higher consumption of UPFs increased the risk of a gain in BMI, waist circumference, and body fat of 5% or more during the follow-up period (median of 5.6 years) [59]. A cohort study with civil servants in Brazil indicated that UPF consumption was associated with an increased relative risk of 27% (95% CI: 1.07–1.50) weight gain and 33% increased relative risk (95% CI: 1.12–1.58) for waist-circumference gain [60]. Fazzino et al. [61] conducted a prospective study among 82 individuals without obesity and found that an increased consumption of UPFs in a buffet meal were associated with greater weight gain over the next 12 months. These findings from cohort studies build on the cross-sectional studies by providing evidence of direction of causality for UPF consumption and weight gain.

In adults, results that are primarily from observational studies generally report that consumption of UPFs is associated with an increased risk of hypertension [62], cardiovascular disease [63], type 2 diabetes [64], metabolic syndrome [65], higher risk of overall cancer [66], and all-cause mortality [67]. Many of these studies adjusted for BMI in the main analyses and/or included sensitivity analyses to adjust for BMI, weight gain, physical activity levels, or a family history of the specific health condition, suggesting that high UPF diets increase one’s risk for co-morbidities independent of body weight. In most of these studies, the participants with the highest UPF consumption also consumed diets of lower overall quality. Participants that consumed the most UPFs had higher intakes of sugar, saturated fats, and salt, but lower and/or inadequate intake of fiber and micronutrients compared to those that had consumed fewer UPF products. Therefore, it is plausible that the consumption of UPFs are associated with many of today’s leading chronic diseases, since poor diet quality is associated with all of the mentioned health conditions [68, 69]. However, there are also a number of possible biological mechanisms unique to UPF consumption, in addition to poor diet quality, that potentially explain some of their effects on increased weight gain and/or chronic disease risk, as discussed further below.

## Ultra-Processed Foods and Health Outcomes in Children and Adolescents

While there is extensive evidence from several systematic reviews and meta-analyses [56, 57, 70–72] linking UPFs to health outcomes in adults, research is more limited in pediatric populations. In 2017–2018, UPFs contributed greater than two-thirds of energy intake among US children and adolescents, a 5.6% increase over the prior 20 years [73]. Additionally, research has found that frequent consumption of UPFs was associated with food addition within overweight children [74]. Together, poor diet quality and excessive caloric intake can contribute to the development of overweight and obesity among children.

Within several prospective studies, higher consumption of UPFs during childhood was associated with more rapid increase in BMI, fat mass index, weight, and waist circumference in adolescence and early adulthood [75–77]. A 2018 systematic review reported that consumption of ultra-processed foods during childhood and adolescence was positively associated with adiposity [80]. Contrarily, several studies have found no association between consumption of ultra-processed foods and weight status or adiposity [78, 79]. Research suggests that null findings may be attributable to factors associated with the etiology of obesity, such as physical activity, genetics, and family lifestyle, which were not assessed within the studies [78, 79]. Given these findings, reducing ultra-processed food consumption among children may reduce the prevalence of overweight and obesity among children; however, a clinical trial within a child population is needed.

In addition to weight and adiposity outcomes, several studies have found a positive association between greater UPF consumption and blood lipids. A Brazilian cohort found that children with the highest consumption of UPFs at 3–4 years of age had greater total cholesterol and triglycerides 4 years later, and another Brazilian cohort found elevated LDL cholesterol and triglyceride levels several years later [80, 81]. Additionally, a study of 210 adolescents in Brazil reported that high consumption of UPFs was associated with the prevalence of metabolic syndrome, a cluster of risk factors that increase the risk for cardiovascular disease, stroke, and diabetes [82]. However, a Spanish cross-sectional study found no significant associations between ultra-processed consumption and HDL or triglyceride levels [83]. This null finding may be attributable to lipids being measured in only a subset of the study population, which could impact lipid levels.

In sum, current research suggests that consumption of UPFs may lead to excessive calorie intake, weight gain, and abnormal blood lipids in the short term and progress into long-term health consequences in adulthood. Given that lifelong dietary patterns develop from childhood and continue into adulthood [84, 85], efforts should be taken to reduce children’s exposure and consumption of energy dense and nutritionally poorer ultra-processed foods.

## Potential Mechanisms of How Ultra-Processed Foods May Increase Weight Gain and Chronic Disease Risk

UPFs induce high glycemic responses, but have low satiety potential [86]. One well-controlled randomized crossover study indicated that the consumption of UPFs led to increased energy intake and weight gain relative to whole foods [87]. In this study, 20 adults ate a diet consisting of mostly UPFs (~ 80% of calories were from UPFs) and an alternate diet of mostly whole grains and unprocessed foods for 2 weeks each. The researchers matched the diets for total energy intake and macronutrient, but the UPF diet resulted in a higher proportion of added total sugar (∼54% versus 1%, respectively), insoluble to total fiber (∼16% versus 77%, respectively), saturated to total fat (∼34% versus 19%). A key finding from the study was that during the UPF phase of the study, the participants consumed 500 kcal/day more than the alternate diet and the participants gained 0.9 ± 0.3 kg (*P* = 0.009) during the UPF diet and lost 0.9 ± 0.3 kg (*P* = 0.007) during the unprocessed diet. The changes in participants’ hunger related hormones (pancreatic peptide YY and ghrelin) during the UPF dietary phase may explain the increased ad libitum energy intake [87]. It has also been suggested that specific features from food processing, such as the inclusion of additives and alteration of the food matrix makes the foods have a softer texture for less chewing and amplifies sensory properties, which delays satiety signaling, and thereby results in an overconsumption of foods [58]. The higher sugar, fat, and salt content in UPFs makes them more hyperpalatable, which in turn could result in a healthy, nutrient-dense diet being displaced with empty calories and a lower-quality diet that results in weight gain [88].

UPFs also have been reported to contribute to a gut environment that selects microbes that are associated with inflammatory disease [89]. The modification of the food matrix often changes the fiber and fat content of the foods, which influences the microbiota composition and bacteria–host interactions [88]. Minimally processed or natural foods have intact fibrous cell walls that provide a substrate for fiber-degrading bacteria in the colon and ensure a slow release of nutrients along the digestive tract [90]. However, the nutrients in UPFs are largely acellular, which instead results in an environment that promotes inflammatory gut microbiota that are associated with various cardiometabolic conditions [89]. Thus, not only are UPF diets usually low in dietary fiber, but even the way fiber is altered from food processing impacts its effectiveness on promoting a beneficial gut microbiome environment. As reported in an animal model study, the consumption of a high-fiber diet in pigs based on processed, extruded grains reduced bacterial diversity compared to a diet based on unprocessed whole grains [91]. A review of 7 trials indicated that higher UPF diets were the most commonly associated with a reduced abundance of microbes that are linked with beneficial health outcomes and an increased abundance of microbes linked with adverse health outcomes [92].

Another mechanism by which UPF consumption might impact biology or metabolism could involve the endocrine-disrupting chemicals, such as bisphenol A (BPA), often found in the elaborate packaging materials used for UPF products [88]. While the complete mechanisms of BPA remain unknown, there is some evidence that BPA promotes insulin resistance, oxidative stress, inflammation, and adipogenesis, which in turn increases our risk for major CVD conditions, including diabetes, overall and abdominal obesity, and hypertension [93].

Gibney (2019 and 2020) argues that any adverse effects observed from UPFs are due to nutritional factors, rather than the degree to which foods are processed [29, 94]. Findings from the SWAP-MEAT crossover intervention study conducted by our lab group indicated that participants had improvements in several cardiovascular disease risk factors during 8 weeks of consuming alternative plant-based meat products relative to organic animal meats [95]. This was due to the simultaneous decrease in saturated fatty acids and increase in dietary fiber that the plant-based meats provided. It is not clear if the level of food processing increases our risk for weight gain and other chronic diseases independently from the nutritional composition of the foods themselves, since most UPFs by default are dense in energy and poor in nutritional quality [96].

However, in our SWAP-MEAT study, there was a small but statistically significant decrease in weight on the plant-based meat phase vs. animal meat phase. However, our study was designed to focus on a single substitution of plant-based meat for animal meat. In a cohort study where the overall level of UPF consumption was examined, across all food types, the investigators reported that after controlling for several components of nutritional quality, UPFs were associated with a higher risk of cardiovascular disease [63]. Similarly, another study found an increased association between the consumption of ultra-processed foods and type 2 diabetes among individuals from the NutriNet-Santé cohort after controlling for diet quality and energy intake [64].

## Policies to Reduce Ultra-Processed Food Consumption

Acknowledging the negative associations between health and UPF, a number of countries have begun implementing polices in an effort to reduce UPF consumption, including taxes on sugar sweetened beverages or snacks, front-of-package (FOP) warning labels, setting limits on sodium and trans-fat content in food products, regulations to reduce or ban the marketing of UPFs, and restricting access and promotion of UPFs in schools [97]. Mexico was one of the first countries to rigorously evaluate its tax policy on sweetened beverages and found that purchases of taxed beverages fell by 6% and the reductions from pre-tax trends were highest among lower socioeconomic status household [98]. While taxes on UPF foods are effective for reducing the sales of such products, a particular gap in fiscal policy is the absence of subsidies or incentives that promote the purchase of healthier foods [97]. There is evidence that food taxes on unhealthy foods combined with subsidies to purchase healthier foods improves the population’s diet quality and health outcomes [99, 100]. However, implementing more forceful policies remains a challenge, since the food industry and other stakeholders are resistant to reducing UPF consumption or are making efforts to undermine public actions to improve health [97, 101].

## Conclusions

Defining the extent of food processing that may be associated with negative health outcomes remains a challenge for the field. Various types of processing remain an integral aspect of providing a safe food system. While the NOVA classification remains the most frequently used method of categorizing foods by level of processing, emerging classification systems seek to build on the limitations of the NOVA classification to provide a more accurate assessment of processing. Epidemiological research suggests that UPF consumption is pervasive and contributes a substantial amount of daily total energy intake in individuals around the world. There has been an observed ecological trend that countries with higher UPF consumption generally have a higher obesity prevalence. However, this trend is not observed in all countries, and differences may be attributable to sociodemographic characteristics or other related factors.

Despite the growing literature documenting the potential increase in weight gain and adverse health outcomes in children, adolescents, and adults from the consumption of UPFs, there has only been one randomized clinical trial specifically assessing the effects of UPF consumption [87]. Therefore, most of what is known about UPFs is based on observational cohort studies, limiting conclusions to associations rather than causation. Several plausible mechanisms including increased energy intake, changes to the gut microbiome, alterations in the gut–brain satiety signaling, and hormonal effects have been proposed as plausible explanations of the observed associations between UPF and both weight gain and risk for chronic disease development. Further research to examine the causal effect of consuming UPFs on weight gain and adverse health outcomes is warranted. Given that UPFs tend to be more energy-dense than nutrient-dense, cautionary recommendations to limit UPF consumption would be unlikely to lead to any additional risk or harm, and would more plausibly lead to a nutritional benefit. Therefore, while awaiting further research, recommendations to limit or restrict UPF consumption would likely lead to more benefit than harm.

## Data Availability

Not applicable.
